# Seasonal records of benthic macroinvertebrates in a stream on the eastern edge of the Iguaçu National Park, Brazil

**DOI:** 10.3897/BDJ.8.e54754

**Published:** 2020-07-14

**Authors:** Jhenifer Simões Santos, Luciano Lazzarini Wolff, Lucíola Thais Baldan, Ana Tereza Bittencourt Guimarães

**Affiliations:** 1 Western Paraná State University, Biological Investigations Laboratory, Cascavel, Brazil Western Paraná State University, Biological Investigations Laboratory Cascavel Brazil; 2 Federal University of Paraná, Water Quality and Limnology Laboratory, Palotina, Brazil Federal University of Paraná, Water Quality and Limnology Laboratory Palotina Brazil

**Keywords:** Macroinvertebrates records, biodiversity, aquatic insects, seasonal precipitation, INP, forest edge stream, surrounding management

## Abstract

**Background**

Iguaçu National Park (INP) is known worldwide due to Iguaçu Waterfalls, being considered a World Natural Heritage by UNESCO. The INP is one of the last large forested extensions of inland Brazil that provides protection to the Atlantic Forest, one of the world’s biodiversity hotspots. However, its Natural Heritage status has been threatened by the construction and operation of the Baixo Iguaçu dam, agricultural and urban impacts on its boundaries and the increasing interest of the Brazilian government in re-opening of the “Colono road”, an old illegal road that crossed the interior of the park. Indeed, since benthic macroinvertebrates have been widely used for the environmental assessment of streams, records and abundance of their taxa under different seasonal periods may provide an additional dataset for biomonitoring of hydrographic systems in the face of current anthropogenic impacts on the INP boundaries and other similar streams on forest edges.

**New information**

In this study, we improved the sampling design of benthic macroinvertebrates and provided seasonal records covering distinct precipitation/temperature periods between 2016 and 2017 of a stream on the eastern edge of the Iguaçu National Park, Brazil. The records total 2,840 individuals distributed in 88 different taxa. The most abundant taxa were the Diptera subfamilies, Chironominae (n = 1,487) and Tanypodinae (n = 256), besides the *Heterelmis* genus (n = 152, Elmidae; Coleoptera). Diptera was the richest order in number of families (n = 8), while Leptophlebiidae (Ephemeroptera) was the richest taxonomic family in number of genera (n = 11). *Aegla* (Crustacea) and the Insecta genera, *Heterelmis, Hexacylloepus, Noelmis, Phylloicus* and *Thraulodes*, were recorded through all samplings. Twenty-five genera of Ephemeroptera, Plecoptera, Trichoptera (EPT) and Odonata were recorded during intermediate precipitation/temperature periods. Twenty-one of them were recorded in May 2016, with five genera standing out in abundance (*Hydrosmilodon, Anacroneuria, Argia, Coryphaeschna, Americabaetis*) and four (*Needhamella, Tikuna, Simothraulopsis, Neocordulia*) in December 2016. Four general taxa were exclusive of the lower precipitation/temperature period (August 2016), standing out in abundance were the *Oxystigma* (Odonata) and *Corydalus* (Megaloptera) genera. In March 2017 (higher precipitation/temperature period), four exclusive taxa were recorded, amongst them, the *Chimarra* (Trichoptera) genus. Furthermore, seasonal records demonstrated higher occurrences and abundance of macroinvertebrates during the intermediate and lower precipitation/temperature periods, besides a varied taxa composition throughout the year, with the presence of both sensitive and tolerant groups to environmental impacts. Our findings suggest that the number and composition of the local-stream macrobenthic fauna were influenced by the seasonal climatic regime. These changes should be considered in the limnological monitoring developed on the hydrographic systems of INP eastern edges to improve the assessment of environmental quality under different local seasonal conditions.

## Introduction

Behind the scenic beauty provided by the Waterfalls, the Iguaçu National Park (INP) represents the largest forest remnant in southern Brazil for biodiversity protection of the inland Atlantic Forest, one of the most biodiverse and threatened biomes (Marchese 2015). The INP has an extension of sub-tropical broadleaf forest of somewhat over 185 thousand hectares (ICMBio 2018). It is comprised of a transition zone between Mixed Ombrophilous Forest and Semideciduous Seasonal Forest, two distinct Atlantic Forest physiognomies (Joly et al. 1999). There are 761 angiosperms, about 250 of them being arboreal species (ICMBio 2018) and 84 mammal species, including some endangered ones, such as the jaguar, puma and tapir (Brocardo et al. 2019). The INP’s terrestrial fauna is also estimated to comprise more than 550 birds, 635 butterflies, 79 reptiles and 55 amphibians (WWF-Brazil 2014) and for aquatic fauna, 18 species of stream fish (Delariva et al. 2018) and 193 taxa of benthic macroinvertebrates at hydrographic systems that flow to below the Waterfall (Moretto and Pujarra 2017) are reported.

Despite internal conservation, INP’s Natural Heritage status, granted by UNESCO in 1986, has been threatened by the construction and operation of the Baixo Iguaçu dam (UNESCO 2012, UNESCO 2019), besides the impacts of agriculture, urbanism and highways (i.e. BR163 and BR277) on its eastern boundaries (Fig. 1). Nowadays, the INP eastern edges are surrounded by a landscape that has been heavily altered by logging, intensification of industrial and small-scale agriculture and silviculture for pulp and paper. Nevertheless, there are no buffer strips or adequate land use planning in adjacent areas to minimise the edge effects on the INP eastern boundaries. Other threats to INP consist of agricultural encroachments, poaching and plant extractions, beyond the increasing interest by the Brazilian government (with the bills PL 984/2019 and PLC 61/2013 in process at the National Congress) to re-open the “Colono road”, an antique illegal road that crossed the interior of the park, from Serranópolis do Iguaçu to Capanema municipalities (UNESCO 2019).

Regarding the hydrography, the INP stands out for hosting the unique entirely preserved hydrographic basin of Paraná State, the Floriano River basin. Other micro-basins, such as those of Silva Jardim and São João Rivers, which flow across the park, are partially preserved (ICMBio 2018). Along the INP eastern edge are the Gonçalves Dias River main channel and its tributary Jumelo Stream that flows to Iguaçu River and, with the others, contribute to the discharge from the Waterfall. This hydrographic system is on the border between the right bank preserved by the INP and the left bank subjected to urban, agricultural and industrial influences. Despite antagonism between preservation and impacts, only recently the fish fauna of this system has been studied (Neves et al. 2015, Delariva et al. 2018) and no benthic macroinvertebrates record has been made.

Benthic macroinvertebrates constitute a polyphyletic clustering of organisms, which colonise bottom substrates and dwell part or the whole of their lifetimes in aquatic ecosystems (Rosenberg and Resh 1993). Platyhelminthes, Mollusca, Annelida, Crustacea and many immature Insecta forms stand out in richness, abundance, lifestyles and levels of sensitivity to environmental stressors (Chang et al. 2013). For these reasons, macroinvertebrates have long been used as model organisms for stream biomonitoring (Baptista 2008, Buss et al. 2015). Therefore, records of macroinvertebrates allow long-term analyses of changes in environment quality that may reflect in a historical process of stream degradation, as organic/industrial pollution, discharge variations, silting and others (Hepp et al. 2010, Wright and Ryan 2016, Castro et al. 2018).

In this context, seasonal records of benthic macroinvertebrates, along with measurements of water physicochemical parameters in local hydrographic systems, can contribute to the upgrading of INP’s limnological monitoring programme. This programme is a condition imposed by the ICMBio (environment sector responsible by the INP administration) for the Baixo Iguaçu dam operation. Therefore, the monitoring may identify livestock activities and dumping of wastewater or even the dam impacts on Iguaçu River’s tributaries (ICMBio 2018, UNESCO 2019). Additionally, since seasonality may imply the structuring of the macroinvertebrate community, changing their habitat conditions and food availability (Linares et al. 2013), seasonal records of macroinvertebrates may provide a dataset to verify the effects of the interaction between local anthropogenic impacts and seasonal influences.

Seasonal climatic changes may indirectly affect the abundance of benthic macroinvertebrates on their habitat-substrates. For example, rainiest periods cause additional runoff and increasing river discharge, which may intensify macroinvertebrates drifting (Callisto and Goulart 2005). These effects might be substantially heaviest on the more impermeable catchments and on invertebrates that dwell in sandy microhabitats, which are more easily carried downstream (Allan 2004). On the other hand, drier and colder periods may promote the macroinvertebrates colonisation as a function of habitat stabilisation and dissolved oxygen enrichment into the water (Kaller and Kelso 2006, Carvalho et al. 2008). Therefore, it is important to consider the seasonal influences for improving stream monitoring based on macroinvertebrates, since their occurrence and abundance in different habitats may change according to each period of environmental variation.

The INP region and the western part of Paraná State has a history of changes, over the annual period, for their precipitation and temperature regimes. There is no extreme drought or flooding, but a contrast between periods of lower and higher rainfall/temperature, alternated by transitory climatic conditions (Carvalho and Stipp 2004). In this context, it is expected that streams of this region present over-year variations on their flows and water temperature, implying seasonal shifts on macroinvertebrates numbers and composition. It might result in macroinvertebrates increasing during intermediate and low precipitation/temperature and the opposite under contrasting conditions. Therefore, it is suggested that stream diagnoses could depend on the seasonal periods, being unable to reflect entire conditions if made in only one climatic phase.

In the face of that, we provide seasonal records of benthic macroinvertebrates between 2016 and 2017 in a stream on the eastern edge of the Iguaçu National Park, southern Brazil. The Jumelo Stream is one of the sampling sites of a broader project that aims to understand the ecology of macroinvertebrates in rural and forested streams in the region. In this sense, we also recorded the water physicochemical parameters and the taxon abundance categories that may contribute to a local limnological monitoring programme. Our purpose was to discuss seasonal records in an associative way with the habitat characteristics and environmental sensitivity of macroinvertebrates. Implications of the surrounding management were also discussed for effective conservation of macroinvertebrates in local stream systems.

## Material and Methods

### Study area

**Figure 3.**
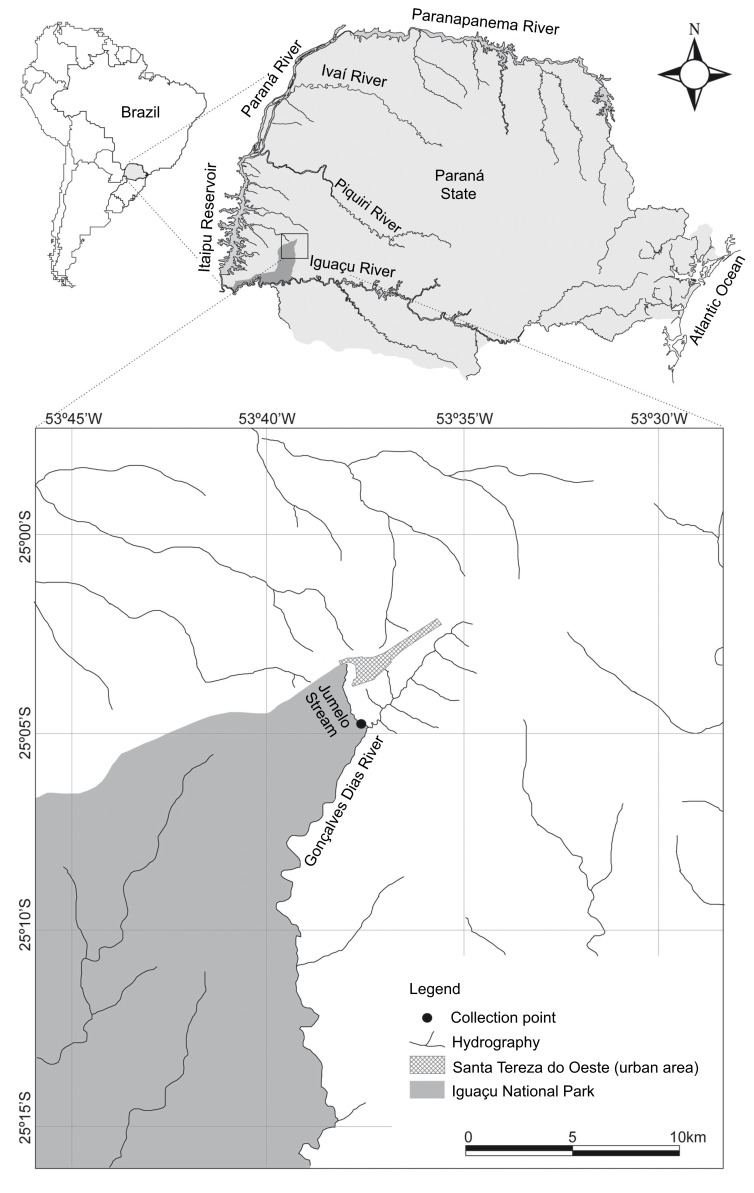
Sampling point (black circle) in the Jumelo Stream. Source: Adapted from Neves et al. (2015).

Sampling was carried out between May 2016 and March 2017, comprising periods of intermediate precipitation and decreasing temperature (May 2016); lower precipitation and temperature (August 2016); higher precipitation and increasing temperature (December 2016) and higher precipitation and temperature (March 2017) for the region (Fig. 2). Hereafter, May and December 2016 are reported as intermediate precipitation/temperature periods, according to Carvalho and Stipp (2004), which define the Apr/May/Jun and Oct/Nov/Dec quarters as periods of climatic transition for the region. The Jumelo Stream is a second-order river of the lower Iguaçu River basin, Western Paraná State, Brazil (Fig. 3). This stream begins at the peri-urban area of the Santa Tereza do Oeste municipality (25°04'46.62" S, 53°37'26.42'' W) and flows in a southern direction, bordering the INP eastern edge. The Jumelo Stream micro-basin has 62% of its area covered by vegetation (mainly because it is part of the INP), 25% by agricultural area and 13% by urban area (Delariva et al. 2018). The right margin of the stream borders the INP and has greater vegetation regeneration (Fig. 4a, b). On the left margin, there is a narrowed riparian vegetation, less than 10 m wide and less regeneration (Fig. 4c). Furthermore, its surroundings consist of a farm with pasture and agricultural uses (Fig. 4d).

### Sampling of macroinvertebrates and laboratory procedures

Sampling of macroinvertebrate fauna was carried out in triplicate on the following substrate categories: fine (sand/clay < 5 mm and gravel of 5-15 mm in diameter), coarse (pebbles of 25-50 mm and cobbles > 50 mm) and leaf litter (leaves and branches of riparian vegetation). Collection was performed using a Surber sampler (0.5 mm mesh), totalling 36 samples. The sampling design attempted to distance the samples more than 5 m from each other, thus providing seasonal records with greater dataset independence. The entire sampling process and laboratory procedures performed with macroinvertebrates are in accordance with the methodology described in the protocol for sampling and preparing benthic macroinvertebrates samples (Santos et al. 2020). All recorded macroinvertebrates were identified in order/suborder, family/subfamily or genera levels (Mugnai et al. 2010, Dias et al. 2006, Salles et al. 2004, Hamada and Silva 2014, Pes et al. 2005, Passos et al. 2007, Trivinho-Strixino and Strixino 1995, Costa et al. 2004), except Nematomorpha phylum. They are available for study in the didactic collection of the Laboratory of Water Quality and Limnology of the Federal University of Paraná (LaQal) - UFPR/Palotina.

### Water physical and chemical characterisation

Water physical and chemical parameters were measured at each substrate category of sampling. The data obtained by a multiparameter equipment for water analysis (HORIBA^®^) were: pH (hydrogen potential), water temperature (^o^C), conductivity (µS.cm^-1^), turbidity (NTU), dissolved oxygen (mg.l^-1^) and total dissolved solids (ml.l^-1^). To verify significant variations amongst the sampling periods in the water physical and chemical variables, a One-way ANOVA was performed. Subsequently, the HSD-Tukey multiple comparison test was applied to determine which averages differed. Analysis were performed on the software Statistica 7.0 (Statsoft 2004).

## Results

The most variations in the water physical and chemical parameters were found for water temperature, which increased significantly from May 2016 to March 2017 (Table 1), reflecting the passage from intermediate/lower temperature (autumn/winter/spring) to higher temperature (summer) periods. Other water variables showed significant differences, for example, pH and turbidity were slightly higher in March 2017 and May/August 2016, respectively, but without any seasonal patterns of changes.

A total of 2,840 macroinvertebrate individuals were collected, comprising 88 different taxa in five phyla, six classes, 17 orders/suborders, 46 families/subfamilies and 57 genera (Table 2). The Diptera subfamilies, Chironominae (n = 1487) and Tanypodinae (n = 256), the *Heterelmis* genus (n = 152, Elmidae; Coleoptera) and the Naididae family (n = 120, Oligochaeta) were the most abundant taxa. Insects stood out, with Diptera being the richest order in number of families (n = 8), followed by Coleoptera (n = 7), Odonata (n = 7) and Trichoptera (n = 6). The richest taxonomic family was Leptophlebiidae (Ephemeroptera) with 11 genera, followed by Baetidae (Ephemeroptera) and Elmidae (Coleoptera) with six genera each and Gomphidae (Odonata) with five genera. Platyhelminthes and Mollusca were represented by only one taxon with only one individual each (Dugesiidae and Sphaeriidae, respectively), while Nematomorpha (n = 2) was the only taxon identified in the high taxonomic level of phylum.

Six genera were common to the four sampling periods (Aeglidae: *Aegla*; Elmidae: *Heterelmis, Hexacylloepus, Noelmis*; Calamoceratidae: *Phylloicus*; Leptophlebiidae: *Thraulodes*) (Table 2). Twenty-five genera of EPT and Odonata were recorded exclusively in the intermediate precipitation/temperature periods. Twenty-one of them, standing out in abundance *Hydrosmilodon, Anacroneuria, Argia, Coryphaeschna* and *Americabaetis*, were recorded in May 2016 and four (*Needhamella, Tikuna, Simothraulopsis, Neocordulia*) in December 2016. Sixty-four taxa, standing out in abundance the *Heterelmis, Hexacylloepus* (Coleoptera), *Phylloicus* (Trichoptera), *Heteragrion* (Odonata) and *Smicridea* (Trichoptera) genera, were recorded in May 2016, while 41 different taxa were recorded in December 2016. In the lower precipitation/temperature period (August 2016), 38 taxa were recorded, five of them exclusive, with higher abundance for the *Oxystigma* (Odonata) and *Corydalus* (Megaloptera) genera. In the higher precipitation/temperature period (March 2017), only 26 taxa were recorded, with four of them exclusive, including the genus *Chimarra* (Trichoptera).

## Discussion

Chironomidae was the most abundant family collected, as found in other aquatic systems (Batista et al. 2010, Barbola et al. 2011). Chironomids can colonise preserved and impacted environments, which make the mixed environmental conditions of Jumelo Stream propitious to their success. In this group, the Chironominae subfamily tends to be predominant (Sanseverino and Nessimian 2008). Its high abundance might be related to its wide distribution in stream systems and their position in basal trophic levels (Sanseverino and Nessimian 2008). The second subfamily ranked in abundance, Tanypodinae, could benefit from the high abundance of other chironomids due its predatory habit (Rieradevall and Brooks 2001). *Heterelmis* (Coleoptera: Elmidae), the third most abundant taxon, occurred with at least ten individuals in all the samplings. This genus is predominant amongst other Elmidae (Arias-Díaz et al. 2007) and is typically found in leaf litter and rocky substrate microhabitats (Cooke et al. 2015, Braun et al. 2014).

Diptera, Coleoptera, Odonata and Trichoptera were the richest orders in number of families. These orders are typical in studies of benthic macroinvertebrates (Hepp et al. 2010, Moretto and Pujarra 2017, Restello et al. 2020). They show, in this sequence, a decrease in their tolerance to anthropogenic impacts (Chang et al. 2013). Naididae (Oligochaeta) and *Aegla*, which were recorded in all samples, are tolerant to impacts (Goulart and Callisto 2003) and foraging in sandy and leaf litter microhabitats, respectively. The low occurrence of other taxa as Platyhelminthes (Dugesiidae), Nematomorpha and Mollusca (Sphaeriidae) may be related to the dominance of Insecta.

Leptophlebiidae (Ephemeroptera) was the richest family in number of genera. *Thraulodes* was recorded in all samplings and showed preference for coarse substrate, according to Shimano et al. (2012). *Miroculis* and the other genera occurred at lowest abundance categories and only in intermediate and lower precipitation/temperature periods, corroborating with Shimano et al. (2012). These authors also reported that low rainfall enables *Miroculis* to colonise rocky substrates. *Thraulodes* and *Farrodes* were the only ones to occur during the higher precipitation/temperature period (March 2017). Although we were not able to test this here, these findings suggest a hypothesis of greater downstream dislodgement of Ephemeroptera genera during higher flows within higher precipitation periods. Moreover, *Baetodes* (Baetidae) was amongst the only three genera of Ephemeroptera also recorded in the higher precipitation/temperature period. Other genera of Baetidae were represented by *Americabaetis, Cloeodes, Cryptonympha, Moribaetis* and *Camelobaetidius*. Siegloch et al. (2008) reported the first three and *Baetodes* in relatively preserved streams. According to Edmunds et al. 1976, *Baetodes* occurs in removed riparian vegetation streams, where solar irradiation allows algae growing and grazing. Otherwise, Dominguez et al. 2006 associate *Americabaetis* with preserved riparian vegetation, but it was also found in impacted environments (Castro et al. 2018). Therefore, records of Ephemeroptera revealed genera with distinct levels of environmental sensitivity and ability to resist the increase in stream flow.

Trichoptera are sensitive to impacts and were also amongst the richest in number of families. Hydropsychidae presented two genera. *Smicridea* was more abundant than *Macronema* and exclusive in intermediate and lower precipitation/temperature periods. Corroborating Spies and Froehlich (2009), *Smicridea* was also recorded in rocky microhabitats. *Phylloicus* (Calamoceratidae) occurred in all the samplings, but was more abundant in intermediate precipitation/temperature periods. This genus was recorded on leaf litter substrate, as registered by Fidelis et al. (2008). *Chimarra* (Philopotamidae) was recorded only in the higher precipitation/temperature period. Unlike *Phylloicus, Chimarra* is not an indicator of preserved sites (Henriques-Oliveira et al. 2015). Plecoptera, also sensitive to impacts, was represented by Perlidae and Gripopterygidae. In Perlidae, *Anacroneuria*, recorded only in May 2016 (intermediate), shows predator behaviour associated with leaf litter microhabitats (Avelino-Capistrano et al. 2017), but here it was exclusive in coarse substrates. Regarding Gripopterygidae, *Paragripopteryx* was recorded in higher precipitation/temperature and other periods, while *Gripopteryx* was recorded in intermediate (May 2016) and *Tupiperla* was recorded in intermediate and lower precipitation/temperature periods. These genera occur in riffles with high levels of dissolved oxygen (Bispo et al. 2002) and may have benefited from the lowest water temperatures.

Odonata is amongst the most tolerant macroinvertebrates. *Progomphus* (Gomphidae), recorded in intermediate and higher precipitation/temperature periods, is adapted to burrowing itself into sandy substrate where it ambushes prey (Kikuchi and Uieda 2005). Unlike most Odonata recognised for their tolerance, *Heteragrion* (Megapodagrionidae), which was abundant and exclusive in intermediate and lower precipitation/temperature periods, is sensitive to environmental deterioration (Ferreira-Peruquetti and Marco-Jr 2002). *Oxystigma* was abundant and exclusively found in the lower precipitation/temperature period (August 2016). This exclusivity may be related to different thermoregulatory strategies in Odonata (Carvalho et al. 2013). Regarding Coleoptera, *Heterelmis*, *Hexacylloepus, Noelmis* (Elmidae), as the Psephenidae family, were recorded in all the samplings. Elmidae is abundant and a generalist found in different habitats (Braun et al. 2014). Psephenidae has dorsoventrally flattened body, adapted to fast currents, and is an indicator of good environmental quality (Hanson et al. 2010)

In general, although there is no temporal replication to test predictions, macroinvertebrates records were higher in intermediate and lower precipitation/temperature periods. On the other hand, only 26 general taxa occurred in higher precipitation/temperature period (March 2017), which corroborate this exploratory study with the hypothesis of seasonal influences of other authors (Callisto and Goulart 2005, Carvalho et al. 2008). A mechanism that may explain these results is that Jumelo Stream is near to Santa Tereza do Oeste city. It becomes another potential impacting cause, besides agricultural influences on its left side. Therefore, highest local rainfall may quickly overcome the capacity for soil infiltration (Lima 2008) and, in impermeable soils, like those near Jumelo Stream, the surface run-off into the stream occurs suddenly (personal observation). This run-off contributes to silting and increasing the stream turbidity with possible effects on habitat structure and occurrence of macroinvertebrates (Allan 2004). Furthermore, the reduced riparian vegetation on the left margin increase these impacts, contributing to an abrupt increase in discharge and its consequences for macroinvertebrates dislodgement. Increasing in water temperature and local irradiance are other factors of the edge effect on the INP eastern limits, which may affect macroinvertebrates communities (Suga and Tanaka 2012, Carvalho et al. 2013)

The high number of taxa, many of them being sensitive to impacts, highlights the importance of the INP forested areas. Since the right margin is preserved, it provides a mosaic of microhabitat environments in the Jumelo Stream, which support multiple taxa with different levels of sensitivity and habitat preferences. Therefore, it is relevant that terrestrial units of conservation are seen with their potential to extend their conservation objectives to freshwater systems (Suski and Cooke 2006). The surroundings of conservation units should be managed to protect watersheds or substantial reaches of river courses (Saunders et al. 2002), in order to avoid excessive run-off and pollution (Derisio 2017). In the case of Jumelo Stream, management should cover from rainwater containment to land use rules of its surroundings.

## Conclusion

The dataset indicated a diverse fauna, composed of 88 different taxa, with distinct levels of environmental sensitivity. Seasonal records showed higher occurrences, abundance and exclusivities in intermediate and lower precipitation/temperature periods. Lower records in the higher precipitation period (March 2017) may be associated with increased run-off and consequent macroinvertebrates drifting. These findings highlight the importance of the INP forested areas, as well as drawing attention to the need for an adequate management of its surroundings and edges. For the Jumelo Stream and other similar streams located at the forest edge, management is necessary to include buffer zones with specific rules and restrictions of land use to reduce local anthropogenic impacts on aquatic biodiversity. Nevertheless, the results indicate the necessity for considering the seasonal effects on the macroinvertebrates fauna to improve the limnological monitoring of the local hydrographic systems, provided in the INP management plan.

## Figures and Tables

**Figure 1a.**
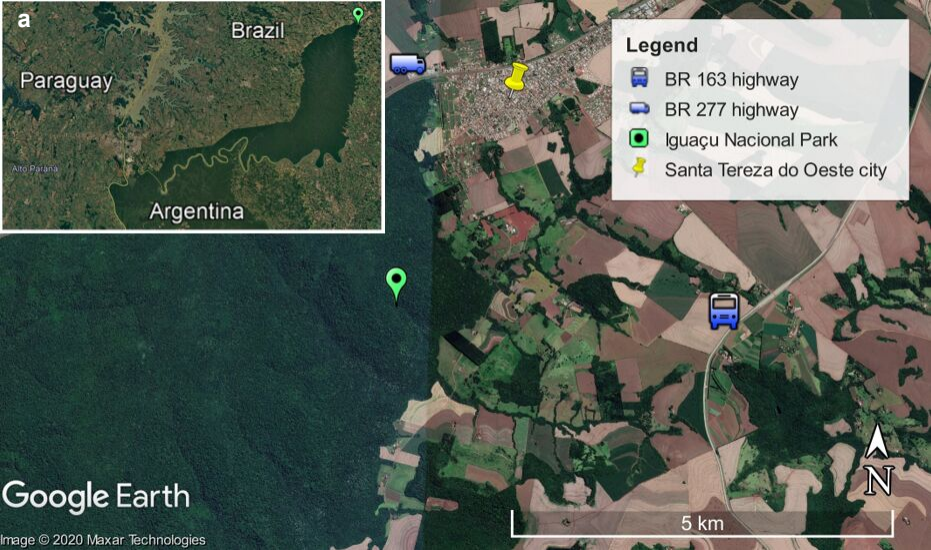
Land use characteristics on sampling site adjacent areas. Source: Google Earth Pro (image date 07/21/2019).

**Figure 1b.**
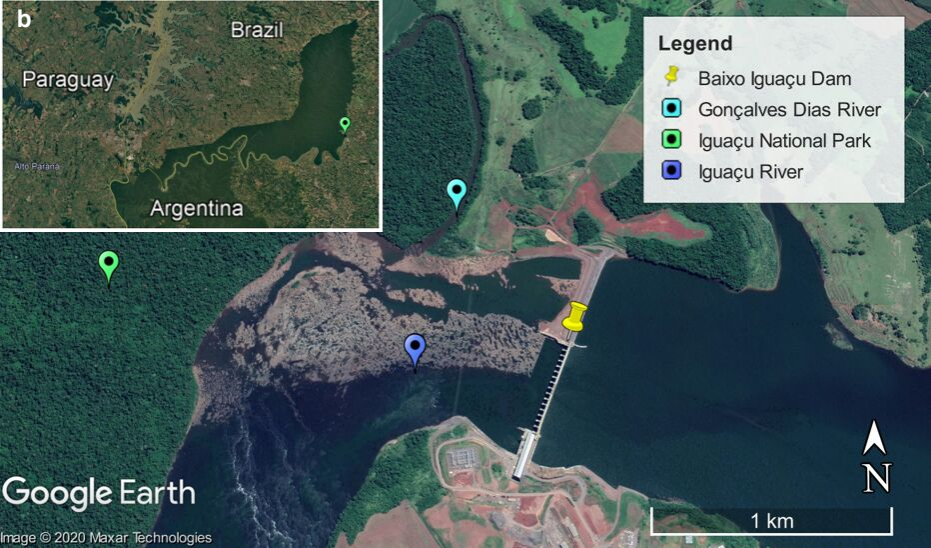
Baixo Iguaçu Dam. Source: Google Earth Pro (image date 10/11/2019).

**Figure 2a.**
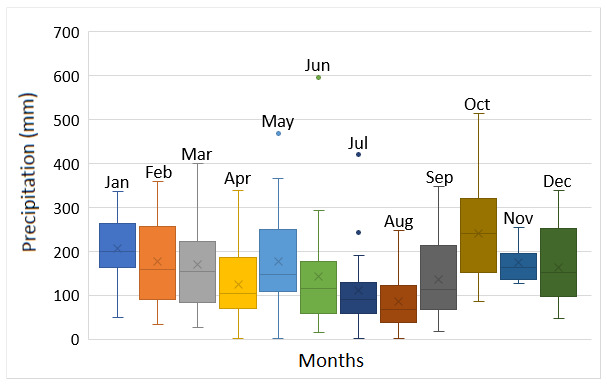
Monthly precipitation: Horizontal line indicates the median, x represents the average, the box includes 50% of the data, from the 25th to the 75th percentile, the vertical lines extend to the 10th and 90th percentiles and the circles are extremes values. Data refer to the period from January 2000 to December 2019. Source: AGUASPARANÁ.

**Figure 2b.**
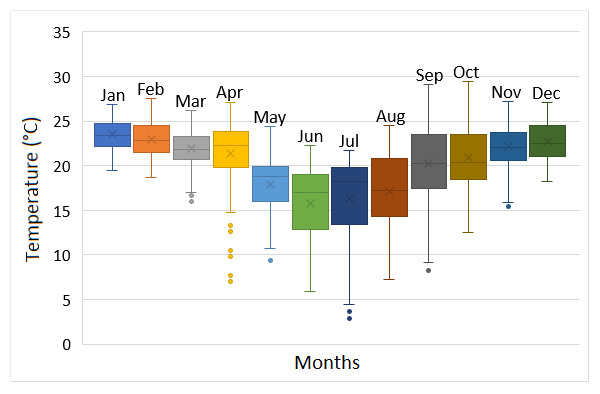
Monthly air temperature. Symbols follow (a). Data refer to the period from January 2016 to December 2019. Source: IAPAR.

**Figure 4a.**
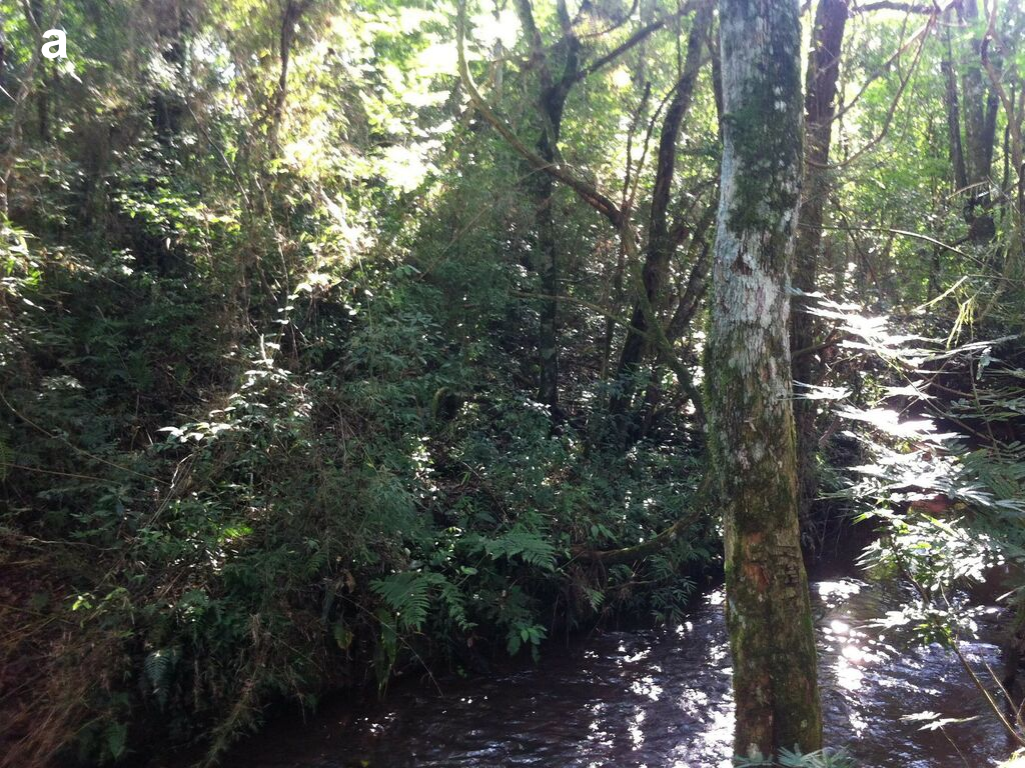
Right margin bordering the INP.

**Figure 4b.**
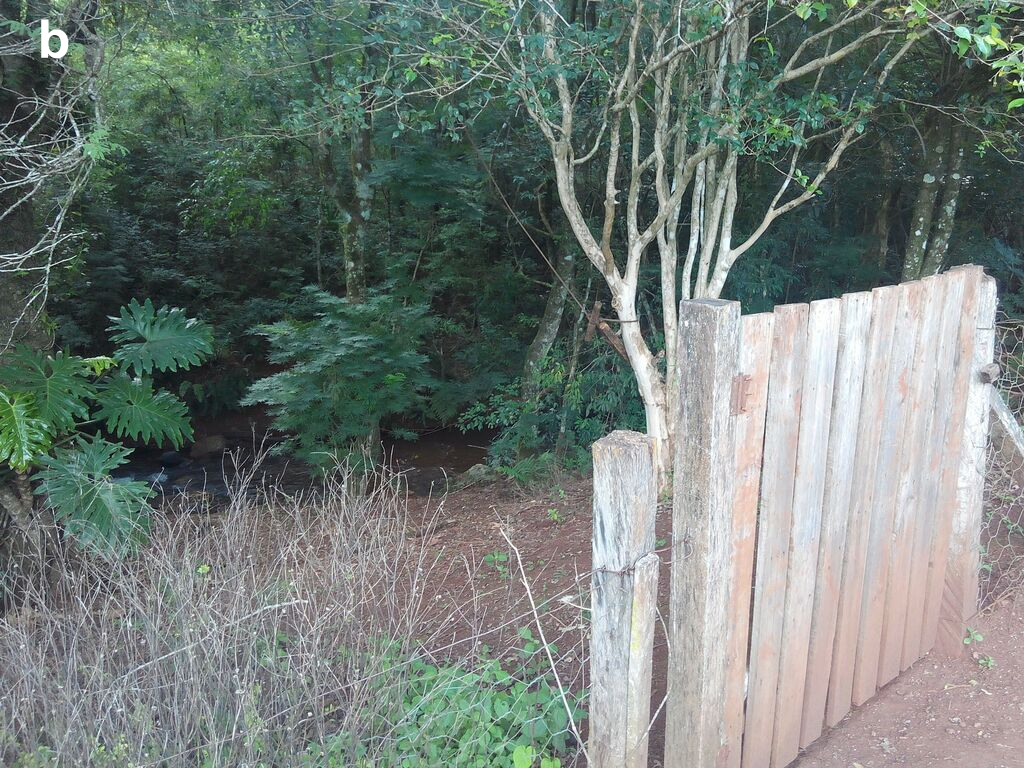
Greater plant regeneration on the right margin than on the left margin.

**Figure 4c.**
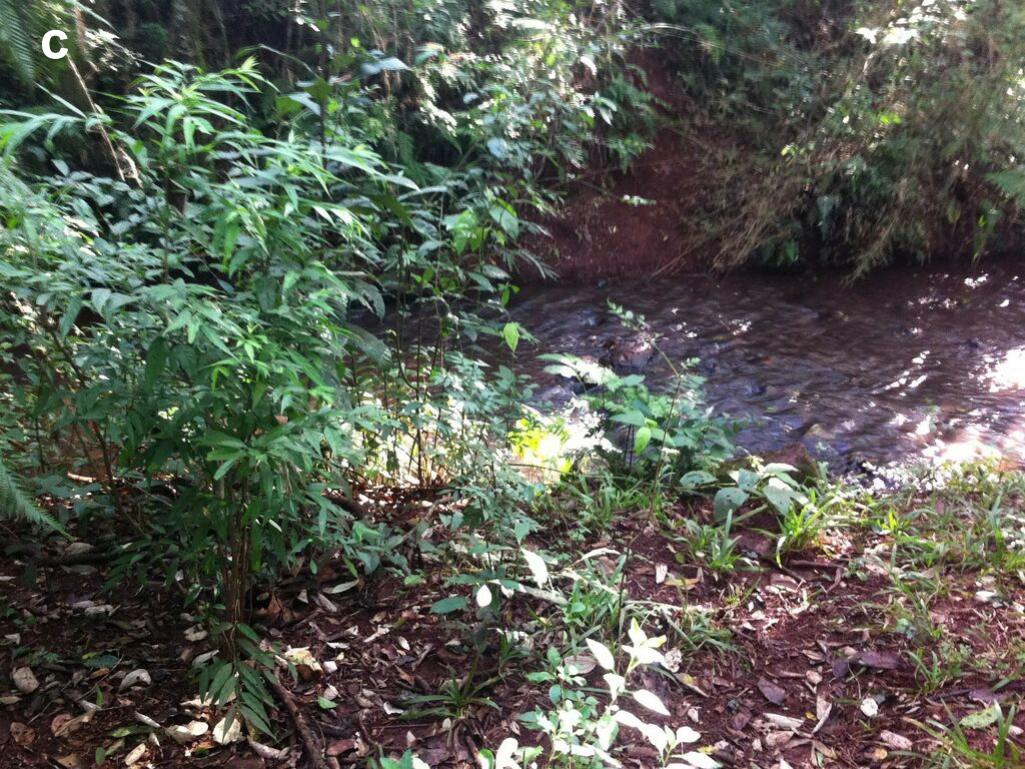
Narrowed riparian vegetation on the left margin.

**Figure 4d.**
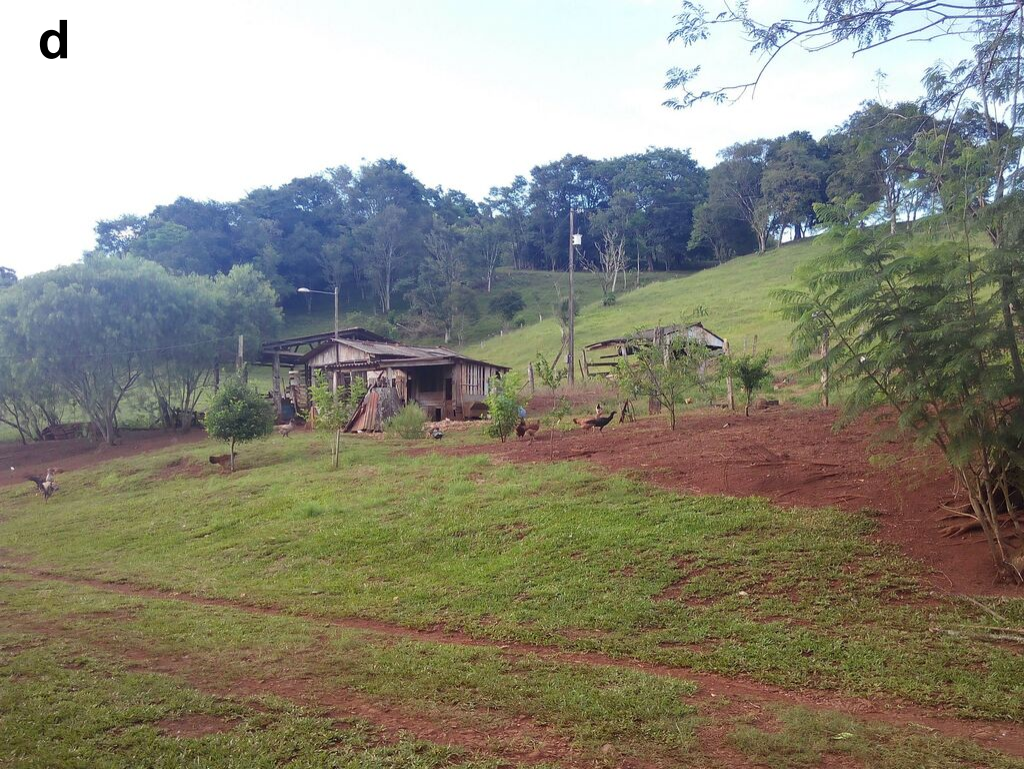
Edifications and grassland use on the left margin.

**Table 1.** Mean ± standard deviation of Jumelo Stream water physical and chemical parameters. DO = dissolved oxygen. Asterisks indicate significance and equal letters indicate statistical similarity amongst seasons. F de Snedecor of One-way ANOVA.

	**May/2016**	**Aug/2016**	**Dec/2016**	**Mar/2017**	**F_(3,32)_**	**p-value**
**Temperature (°C)**	15.75 ± 0.05^d^	19.04 ± 0.37^c^	20.31 ± 0.17^b^	20.76 ± 0.16^a^	824.00	< 0.0001*
**DO (mg.l^-1^)**	9.75 ± 0.29^a^	8.43 ± 1.16^b^	8.47 ± 0.85^b^	9.26 ± 0.87^ab^	5.06	0.006*
**pH (hydrogen potential)**	7.24 ± 0.14^b^	6.82 ± 0.35^c^	6.87 ± 0.49^bc^	8.00 ± 0.18^a^	26.11	< 0.0001*
**Conductivity (µS.cm^-1^)**	0.0287 ± 0.005^c^	0.034 ± 0.002^ab^	0.032 ± 0.003^bc^	0.035 ± 0.0005^a^	8.95	0.0002*
**Turbidity (NTU)**	11.27 ± 2.40^b^	13.45 ± 0.74^a^	9.06 ± 1.08^c^	10.29 ± 0.39^bc^	16.27	< 0.0001*
Total solids (ml.l^-1^)	0.019 ± 0.003^b^	0.022 ± 0.001^a^	0.02 ± 0.001^ab^	0.23 ± 0.00^a^	8.22	< 0.0001*

**Table 2.** Macroinvertebrates records of the Jumelo Stream, eastern edge of the Iguaçu National Park. Abundance classes correspond to: Very low (VL; 1-5), low (L; 6-10), medium (M; 11-50), high (H; 51-100) and very high (VH; > 100) captured individuals.

Phylum/ Class	Order/ Suborder	Family/ Subfamily	Genus	May 2016	Aug 2016	Dec 2016	Mar 2017
Platyhelminthes Turbellaria	Tricladida	Dugesiidae		**VL**			
Nematomorpha					**VL**		
Mollusca Bivalvia	Veneroida	Sphaeriidae			**VL**		
Annelida Oligochaeta	Haplotaxida	Naididae		**M**	**M**	**L**	**M**
Annelida Hirudinea	Rhynchobdellida	Glossiphoniidae		**VL**	**VL**		
Arthropoda Malacostraca	Decapoda	Aeglidae	* Aegla *	**M**	**M**	**M**	**M**
		Atyidae					**VL**
	Amphipoda						**VL**
Arthropoda Insecta	Collembola	Entomobryidae			**VL**	**VL**	
	Ephemeroptera	Baetidae				**L**	
			* Americabaetis *	**VL**		**VL**	
			* Baetodes *	**L**		**VL**	**VL**
			* Camelobaetidius *		**VL**	**VL**	
			* Cloeodes *	**VL**	**VL**	**L**	
			* Cryptonympha *	**VL**		**VL**	
			* Moribaetis *	**VL**			
		Caenidae	* Caenis *	**VL**	**VL**	**VL**	
		Leptohyphidae	* Leptohyphes *	**VL**			
			* Traverhyphes *	**VL**			
			* Tricorythodes *	**VL**			
		Leptophlebiidae				**VL**	
			* Farrodes *	**VL**			**VL**
			* Hagenulopsis *	**VL**			
			* Hydrosmilodon *	**L**			
			* Hylister *	**L**	**VL**		
			* Massartella *	**VL**	**VL**	**VL**	
			* Meridialaris *	**VL**			
			* Miroculis *	**L**	**VL**	**VL**	
			* Needhamella *			**VL**	
			* Simothraulopsis *			**VL**	
			* Thraulodes *	**VL**	**VL**	**VL**	**L**
			* Tikuna *			**VL**	
	Odonata Anisoptera	Aeshnidae		**VL**	**VL**		
			* Coryphaeschna *	**VL**			
		Corduliidae	* Neocordulia *			**VL**	
		Gomphidae		**VL**	**VL**		
			* Progomphus *	**M**		**VL**	**VL**
			* Cacoides *	**VL**			
			* Agriogomphus *	**VL**			
			* Gomphoides *	**VL**			**VL**
			* Phyllocycla *	**VL**			
		Libellulidae		**VL**			
			* Perithemis *	**VL**			
			* Anatya *	**VL**			
			* Brechmorhoga *	**VL**			
			* Macrothemis *	**VL**			
	Odonata Zygoptera					**VL**	
		Calopterygidae	* Mnesarete *	**VL**			
		Coenagrionidae	* Argia *	**L**			
		Megapodagrionidae					**L**
			* Heteragrion *	**M**	**VL**		
			* Oxystigma *		**M**		
	Plecoptera	Gripopterygidae	* Gripopteryx *	**VL**			
			* Paragripopteryx *		**L**	**M**	**VL**
			* Tupiperla *	**M**	**VL**	**M**	
		Perlidae	* Anacroneuria *	**L**			
	Megaloptera	Corydalidae	* Corydalus *		**VL**		
	Coleoptera	Curculionidae				**VL**	
		Dytiscidae		**VL**	**VL**	**VL**	
		Elmidae	* Heterelmis *	**H**	**M**	**M**	**M**
			* Hexacylloepus *	**M**	**M**	**L**	**VL**
			* Macrelmis *	**VL**	**VL**	**VL**	
			* Microcylloepus *	**VL**			
			* Noelmis *	**VL**	**VL**	**VL**	**VL**
			* Phanocerus *	**VL**			
		Hydrophilidae		**VL**			
		Lutrochidae	* Lutrochus *	**VL**	**VL**		**VL**
		Psephenidae		**M**	**VL**	**L**	**VL**
		Ptilodactylidae				**VL**	
	Lepidoptera	Crambidae		**VL**			
	Trichoptera	Calamoceratidae	* Phylloicus *	**M**	**VL**	**L**	**VL**
		Ecnomidae	* Austrotinodes *	**VL**			
		Hydrobiosidae	* Atopsyche *	**VL**			
		Hydropsychidae	* Macronema *			**VL**	**M**
			* Smicridea *	**M**	**L**		
		Leptoceridae	* Nectopsyche *	**VL**	**VL**	**VL**	
			* Oecetis *			**VL**	**VL**
		Philopotamidae	* Chimarra *				**VL**
	Diptera	Ceratopogonidae		**L**	**VL**	**VL**	**VL**
		Chironomidae Chironominae		**VH**	**VH**	**VH**	**M**
		Chironomidae Orthocladiinae		**M**	**M**	**M**	
		Chironomidae Tanypodinae		**H**	**M**	**VH**	**L**
		Empididae		**L**	**VL**	**VL**	**VL**
		Muscidae				**VL**	
		Psychodidae		**VL**	**VL**		
		Simuliidae		**M**	**L**	**L**	**L**
		Stratiomyidae					**VL**
		Tipulidae		**VL**			

## Hosting institution

Western Paraná State University – *Campus* Cascavel, Brazil.
